# Pathophysiology of Work-Related Neuropathies

**DOI:** 10.3390/biomedicines11061745

**Published:** 2023-06-17

**Authors:** Tariq Malik, Ahmed Malik, Alaa Abd-Elsayed

**Affiliations:** Department of Anesthesiology, University of Wisconsin School of Medicine and Public Health, 600 Highland Avenue, B6/319 CSC, Madison, WI 53792-3272, USA; tmm@uchicagomedicine.org (T.M.); ahmedm1@uchicago.edu (A.M.)

**Keywords:** work-related injury, chronic nerve compression injury, nerve damage, entrapment neuropathy, carpal tunnel syndrome, ulnar neuropathy

## Abstract

Work-related injuries are common. The cost of these injuries is around USD 176 billion to USD 350 billion a year. A significant number of work-related injuries involve nerve damage or dysfunction. Injuries may heal with full recovery of function, but those involving nerve damage may result in significant loss of function or very prolonged recovery. While many factors can predispose a person to suffer nerve damage, in most cases, it is a multifactorial issue that involves both intrinsic and extrinsic factors. This makes preventing work-related injuries hard. To date, no evidence-based guidelines are available to clinicians to evaluate work-related nerve dysfunction. While the symptoms range from poor endurance to cramping to clear loss of motor and sensory functions, not all nerves are equally vulnerable. The common risk factors for nerve damage are a superficial location, a long course, an acute change in trajectory along the course, and coursing through tight spaces. The pathophysiology of acute nerve injury is well known, but that of chronic nerve injury is much less well understood. The two most common mechanisms of nerve injury are stretching and compression. Chronic mild to moderate compression is the most common mechanism of nerve injury and it elicits a characteristic response from Schwann cells, which is different from the one when nerve is acutely injured. It is important to gain a better understanding of work-related nerve dysfunction, both from health and from regulatory standpoints. Currently, management depends upon etiology of nerve damage, recovery is often poor if nerves are badly damaged or treatment is not instituted early. This article reviews the current pathophysiology of chronic nerve injury. Chronic nerve injury animal models have contributed a lot to our understanding but it is still not complete. Better understanding of chronic nerve injury pathology will result in identification of novel and more effective targets for pharmacological interventions.

## 1. Introduction

Work-related injuries are common. In 2021, the U.S. Bureau of Labor Statistics reported more than 2.5 million nonfatal injuries at a cost of USD 167 billion to USD 350 billion. A significant number of work-related injuries involve nerve damage or dysfunction. While numerous factors can predispose a person to nerve damage, in most cases it is a multifactorial issue that involves both intrinsic and extrinsic factors. Nerve damage is deemed “work related” in the absence of medical conditions that can cause nerve damage. Evidence-based guidelines are not available to clinicians to evaluate work-related nerve dysfunction. The damage varies from obvious nerve transection from a traumatic injury at work to subclinical damage from repetitive microtraumas over the years that may be difficult to ascribe to work. The symptoms vary from poor endurance to cramping to clear loss of motor and sensory functions. The most common risk factors for nerve damage include a superficial location, a long course, an acute change in trajectory along the course, and coursing through tight spaces. Nerve damage is most commonly caused by stretching or compression. In addition, long incubation periods make work-related injuries difficult to detect and even more difficult to prevent. Nevertheless, it is important to have a better understanding of work-related nerve dysfunction, both from health and from regulatory standpoints.

## 2. Epidemiology

Nerves are most often damaged by compression mechanisms that present as entrapment neuropathy. The median nerve is most often damaged at the wrist. Carpal tunnel syndrome is the most common entrapment neuropathy, with a 3% prevalence in the general population [1], and reaching upward of 15% in occupational settings. Ulnar neuropathy is the next most common, with a prevalence of around 0.6–0.8% and reaching upward of 6% in self-reported questionnaires [2]. Radial nerve compression, while less well known, is seen in numerous professions, especially those that involve prolonged hand loading and repetitive hand gripping. In 2000, Latinovic et al. [3] analyzed the UK General Practice Research Database of 1.83 million patient-years among patients registered with 253 general practices. According to their report, the annual age-standardized rates per 100,000 of new presentations in the primary care of various peripheral entrapment syndromes were as follows: carpal tunnel syndrome, men 87.8/women 192.8; Morton’s metatarsalgia, men 50.2/women 87.5; ulnar neuropathy, men 25.2/women 18.9; meralgia paraesthetica, men 10.7/women 13.2; and radial neuropathy, men 2.97/women 1.42. The presentation of nerve dysfunction was most commonly diagnosed between the ages of 55 and 64 years, except for carpal tunnel syndrome, which was most common in women aged 45–54 years, while radial nerve dysfunction was most frequently seen in men aged 75–84 years (Table 1).

## 3. Risk Factors

A factor that results in narrowing the space around the nerve or stretching the nerve beyond its limits predisposes the nerve to malfunction. While the insult may not be severe, repetitive minor insults can overwhelm the physiological reserves of the nerve, which results in neuropathy. Patients with square-shaped wrists, as defined by an anteroposterior-diameter-over-mediolateral-diameter ratio of more than 0.7, are more likely to develop carpal tunnel syndrome [3]. Diabetes and hypothyroidism predispose people to work-related entrapment neuropathy due to the underlying disease pathology of soft-tissue accumulation [4]. Joint laxity and repeated joint dislocations are commonly found in patients with Ehlers–Danlos syndrome. Ulnar nerve stretch injuries are much more prevalent in Ehlers–Danlos syndrome patients, attributed to ulnar nerve stretching from repeated dislocation at the elbow joint [5].

## 4. Pathophysiology

The common mechanisms of work-related nerve injuries are compression, stretching, and laceration. Loss of function is either from physical disruption as in acute injury or from loss of myelin or axons over time. Axonal damage is followed by Wallerian degeneration. Seddon provided the first description of various nerve injuries in 1943 [6]. He coined the terms “neuropraxia”, “axonotmesis”, and “neurotmesis” to describe the extent of injuries and recovery patterns. In neuropraxia, no disruption of axons or any other surrounding tissue exists, so recovery is quick and complete. Axonotmesis is characterized by damage to the axons, where recovery is slow and may not be complete. Neurotmesis is complete disruption of the nerve. A decade later, Sunderland provided a more detailed account of the types of nerve injury, where nerve damage is assigned a grade based on increasing nerve damage rated as grades I–V, with mixed or combinations of various types of nerve injury described by Mackinnon and Dellon as a separate type of nerve injury, calling it grade VI [7] (Table 2).

Nerve injury when higher than grade 2 is accompanied by Wallerian degeneration. The damaged axons are cleared by inflammatory cells, and so begins the repair process. Schwann cells play a prominent role in nerve injury and repair.

Earlier studies of nerve injuries focused primarily on the visible changes that occur after injury, Wallerian degeneration. The downregulation of myelin-expressing genes in Schwann cells participating in nerve repair led to the idea that the Schwann cell response to nerve injury is to regress into an earlier version of a developmentally immature cell, a cell form that presents in the embryonic stage before the myelin sheath develops [8]. In uninjured nerves, promyelination transcription factors, such as *krox-20* (Egr2), which initiate and maintain myelination, are balanced by factors, such as *c-jun*, which favor meylinolysis. This mutual antagonism between *krox-20* and c-jun modulates the transcription factor *sox-2* within Schwann cells, which determines whether the Schwann cells will stay in an undifferentiated state, which is needed for repair [9].

The discovery of transcription factors, which are not active during myelin sheath formation or during the neuron developmental stage but are found to be upregulated in the injury phase, led to the current theory that phenotypic switching in Schwann cells in response to injury is not merely a regression phenomenon. The *c-Jun* and chromatin modifications involving H3K27 demethylation, H3K27 deacetylation, and H3K4 methylation were among the first transcription factors recognized as necessary in the normal execution of the Schwann cell injury response, even though they play no role in regulating neonatal Schwann cells or myelination [10]. More transcriptional control mechanisms that are only active during the repair phase have since been discovered, such as *Merlin* and *STAT3* [11]. Schwann cells involved in repair after injury express protein molecules, such as Sonic Hedgehog (Shh) and Olig 1, which are also not found in the embryonic stage [12]. Schwann cells also develop properties of an inflammatory cell expressing cytokines attracting macrophages [13]. These series of changes within Schwann cells not only reflect the active nature of the repair but also are seen in other tissues—called direct reprogramming or adaptive cell reprogramming. The discovery of upregulation of epithelial mesenchymal transition (EMT) of stemness genes in Schwann cells makes repair transformation similar to repair cells seen in other tissues [14].

Transforming Schwann cells from a support-type role to a repair cell mode involves two main changes: (1) stopping of myelin sheath formation and (2) sequential activation of processes. The first change involves downregulation of the pro-myelin transcription factor *Egr-2* (Krox20), cholesterol-synthesizing enzymes, and other proteins needed for myelin sheath synthesis and upregulation of factors that express g L1, NCAM, p75NTR, and glial fibrillary acidic protein [15]. The second change involves sequential activation of processes that support repair, including:

- Activation of the immune response, resulting in the production of cytokines, including TNFα, LIF Il-1α, Il-1β, LIF, and MCP-1 that attract macrophages.
- Upregulation of proteins that support neuronal survival and facilitate axonal regeneration (GDNF, Ar-temin, BDNF, NT3, NGF, VEGF, erythropoietin, pleiotrophin, p75NTR, and N-cadherin).
- Structural reorganization as Schwann cells become extremely elongated, developing about threefold longer than myelin and Remak cells. These new cells are descendents of Schwann and Remak cells and revert to their original form following regeneration.
- Breakdown of the myelin sheath by Schwann cells.

The phenotypic switch from a myelin-producing cell to a repair Schwann cell, like all repair cells in the body, depends on environmental signals. As regeneration nears completion and repair cells become redundant, the environmental signals that initiated the phenotypic switch in Schwann cells disappear and return to the myelin and Remak phenotypes. Therefore, the repair cell phase is transient—producing on demand and remaining present as long as needed [16].

Schwann cell changes in response to injury have a temporal and orderly sequence, meaning they do not occur simultaneously. Cytokine production peaks within 1 day after injury, myelin lysis peaks around day 5, the *glial-derived neurotrophic factor* (GDNF) protein peaks around day 7, and the *brain-derived neurotrophic factor* (BDNF) protein peaks within 2–3 weeks. *C-Jun* protein levels begin rising immediately following injury and peak about 10 days following injury. The morphological changes of cell elongation and branching take 4 weeks to fully develop [17].

The extrinsic signals initiating these Schwann cell changes include the following: increased activity of phospholipase A2; increased expression of mRNA for LIF, c-Jun and several other AP1-family members, and c-Jun, IL1, Il-1β, and TNFα protein levels; phosphorylation of ErbB2 neuregulin receptor; p38 MAPK activation; and actin polymerization. These changes occur within hours and well before any structural changes are visible [18]. While Schwann cells or an influx of inflammatory cells hints at the possibility that changes are initiated by signals from the damaged or cut axons, so far no signaling pathways have been identified. What is initiating these changes? The evidence points to neuregulin via the ErbB2 phosphorylation pathway, purines via the protein kinase C or ERK1/2 pathway, and neuron-derived hydrogen peroxide by increasing annexin levels [19,20].

The normal functioning of *c-Jun* is imperative for neuron survival and repair. It is linked with 172 functioning genes and directly regulates several neurotrophic factors, such as GDNF, BDAF, Shh, and Artemin [9]. Many of these genes are involved in injury-induced repair. Although the extracellular signals that activate Schwann cell *c-Jun* after injury are not well known, a number of intracellular signals have been identified. These include the Rac1–MKK7–JNK pathway and mTORC, both of which elevate c-Jun, while histone deacetylase 2 (HDAC2), transcription factors Krox20, and Oct6 have been found to antagonize *c-Jun* expression [21,22].

**Table 2:** Peripheral nerve injury [22].

Seddon	Sunderland	Injury	Recovery	Surgery
Neuropraxia	I	Intact basal lamina, no axonal damage	Complete and immediate	No
	II	Axonal damage, endoneurium intact Wallerian degeneration	Complete but over months	No
Axonotmesis	III	Endo-neural disruption Wallerian degeneration	Partial over months	Maybe
	IV	Endo- and perineural disruption Wallerian degeneration	None	Yes
Neurotmesis	V	Complete nerve disruption Wallerian degeneration	None	Yes
	Mackinson type VI	Combination of the above	Variable	Yes

Maintenance of repair cells during the repair time period, which is often spread over months, is also vital for successful outcomes. Failure to maintain a healthy functioning repair cell population is often a main cause of poor outcomes. *STAT3* is the first transcription factor that was found to play an important role in the long-term survival of Schwann cells in repair mode [11].

Schwann cells remove the myelin sheath predominantly by autodigestion and some by phagocytosis mediated by TAM receptors [16]. Macrophages recruited by Schwann cells not only help remove myelin debris but also stimulate vascular and directional axonal growth. Schwann cell recruitment of macrophages is under strong regulation by the Raf–MEK–ERK mitogen-activated protein kinase [23].

The role of EMT/stemness was initially studied in the context of cancer development. It represents a process of cell reprogramming that explains the transition from an epithelial to a mesenchymal cell form. It is characterized by downregulation of molecules that promote cell-to-cell adhesion to increased capacity for migration, increased capacity for proliferation, and morphological plasticity. The phenomenon is now known to be part of the normal healing process. Activation of EMT is associated with activation of genes present in stem cell states that result in increased stemness of cells, i.e., cells exhibiting more plasticity. Recent papers provide evidence that an EMT-like process is active in the Schwann cells of injured nerves [19,22].

Schwann cells de-differentiate and switch from myelin-maintaining cells to growth-producing factors. The switch occurs either due to injury or from loss of axonal signaling due to damaged axons [24]. Schwann cells are specifically aligned to form longitudinal tube-like structures—bands of Bungner—that help direct regenerating axons toward the distal injured stump. Fibroblasts use similar signaling (ephrinB/EphB2) to bridge the wound gap in the skin, thus producing directional migration during wound healing [25]. Macrophages in the presence of hypoxia, mostly seen in the healing of injured tissues, promote neovascularization by releasing vascular endothelial growth factors. Neovascularization helps promote the directional growth of axons, as interruption of this process disturbs the directional movement of Schwann cells and leads to suboptimal recovery [26].

Our understanding of nerve injury is mostly based on lab models, where nerve injury models are acute and severe in nature. However, most work-related nerve injuries are chronic and mild in nature, which led to the belief that most chronic compressive nerve injuries undergo a mild version of Wallerian degeneration, which is now being realized as not true. Pham and Gupta [27] reviewed the nerve response to chronic compression. Most of the data still come from various animal models of chronic nerve injuries developed to mimic entrapment neuropathies in humans. Such studies have shown that one of the most prominent changes includes alteration of the axon-over-total-fiber-diameter ratio, which increases from 0.6 to 0.8. Limited human studies have revealed thinning of myelin and thickening of the endo-, peri-, and epineurium along with increased vascularity of the endoneurium [28]. A slowing of nerve conduction without axonal loss is due to altered remyelination after demyelination by Schwann cells. This morphological change is characterized by a thinner layer of myelin, a shorter inter-nodal distance, and increased Schmidt–Lanterman incisures along the axon as compared to normal nerves [29]. The rise in the number of Schmidt–Lanterman incisures may represent an increase in metabolic demand secondary to demyelination and remyelination in the chronic nerve injury state. All these changes may also explain the slower conduction velocity across an entrapment point. Animal chronic nerve injury models reveal proliferation of Schwann cells after a chronic nerve injury both at the compression site and in the distal nerve segments. The response is seen at 2 weeks post-injury, and it peaks around 4 weeks, reaching six times the number in the baseline population. This proliferative response of Schwann cells is accompanied by a high level of apoptosis at the same time [30]. These two responses occur in the absence of any axon damage. Downregulation of myelin-associated proteins occurs as part of the Schwann cell demyelination response. This promotes axonal sprouting following chronic nerve injury. This sprouting also occurs in the absence of any axonal damage. Microscopic examination of nerves from the entrapment site reveals a number of small unmyelinated fibers grouped together by non-myelinating Schwann cells into structures called Remak bundles [31]. The DRG of neurons have also been shown to undergo a phenotypic switch [32]. The number of isolectin-B4-binding- and calcitonin-gene-related-peptide-positive neurons increases with a proportional decrease in neurofilament-200-positive neurons—representing an increase in the number of nociceptive neurons and a decrease in proprioceptive neurons. This may be why patients develop pain in entrapment neuropathies. This phenotypic switch is possibly mediated by a growth factor released by Schwann cells, which are then transported upward toward the DRG to induce the change. Glial-cell-derived neurotrophic factor (GDNF) is upregulated in Schwann cells after injury and is a neurotrophic factor for isolectin-B4-binding neurons [33]. This may explain the phenotypic switch. Surprisingly, studies have revealed that axonal integrity is maintained after chronic nerve compression. A chronic nerve compression animal model revealed the slowing of conduction velocity at 2 weeks following injury but no change in the amplitude during compound measurements of action potentials (CMAPs). No evidence of muscle denervation was observed. The neuromuscular junction anatomy remained structurally unchanged. The macrophage response to chronic nerve compression is slow and mild as opposed to an immediate and massive response following an acute nerve injury [34]. Overall, the hallmark of chronic nerve injury is the Schwann cell response in the absence of axonal injury and blood-derived macrophages. The mechanism of Schwann cell injury response in the absence of axonal damage or inflammation is not known. There is a possibility that Schwann cells are mechanosensitive. Schwann cells proliferate swiftly when exposed to shear stress induced by laminar fluid flow, which potentially explains the response seen in entrapment neuropathies [35].

Compartment nerve injury results from external pressure on the nerve that either causes direct damage to the nerve or interrupts the blood flow of major blood vessels or the vasa nervorum. Kashul et al. outlined the diagnostic criteria of entrapment neuropathy in 1977 [36]. Compression most often occurs where nerves pass through an osteo-cartilagenous tunnel or fascial opening. Nerve injury from compression is a multifactorial and complicated issue. Nerve damage depends on the extent of pressure, the duration of pressure, and the dynamic nature of the pressure. An external pressure of less than 30 mmHg causes venous occlusion, in which a pressure above 50 mmHg starts disrupting arterial blood supply and a pressure above 80 produces acute ischemia [37]. Tissue pressure in these ranges has been demonstrated in vivo at the wrist, elbow, and ankle. The axonal transport stops at a pressure above 50 mmHg and signal conduction at a pressure above 70 mmHg. The effect can linger for days even after pressure is released. A sustained pressure of 200–300 mmHg for 2 h produced a conduction block for 3 days in a rabbit vagus nerve model [37]. External pressure on the nerves produces neural swelling that lasts for hours after pressure is released. This is attributed to ischemic damage from external compression, resulting in axonal edema. Both acute and chronic compressions have been shown to cause demyelination. A high pressure of 200 mmHg for 2 h in a rabbit tibial nerve model and a low pressure of 30 mmHg for 7 days in a rat sciatic nerve model produced delayed demyelination weeks after the pressure incident [35,36,37].

Prolonged low-grade compression induces neural edema, which leads to epidural scarring that produces a thickened nerve that compounds the nerve entrapment issue. Fibrosis induced by external compression impairs the free gliding of the nerves due to adhesion formation. This makes smooth movement at the sharp fascial angle or osseocartilaginous tunnels less seamless and, in turn, adds a stretch-induced nerve injury component.

Prolonged compression or ischemia induces demyelination and focal demyelination, which are hallmarks of entrapment neuropathies that produce a segmental block or a slowing of conduction. Persistent compression may eventually lead to axonal loss. Large fibers are much more susceptible to injury from compression than small fibers. However, lately, it has been seen experimentally that not only are small fibers equally vulnerable but also their dysfunction may even precede that of large fibers. Altered myelination and blood flow affect the electrical properties of nerves, including upregulation and downregulation of various ions as well as the expression of novel channels. Neuroinflammation is involved in the generation and maintenance of entrapment neuropathy. Immune cell activation following injury releases inflammatory mediators that damage nerve tissue barriers, causing an influx of mediators. This neuroinflammation contributes to the sensitization of injured and uninjured neuronal tissue, thus contributing to the initiation and maintenance of chronic pain. Glial cell activation is seen after nerve root compression in the spinal cord and with substantial peripheral nerve injuries [38]. This remote neuroinflammation explains the spread of symptoms beyond the affected dermatomes or the innervation territories in patients with nerve damage. Experimentally, mild compression of the nerves evokes an inflammatory response that is confined to the epineurium but still leads to the sensitization of the nervi nervorum, thus inducing spontaneous activity in nociceptive axons.

Central neuronal changes after nerve injury are invariably involved, although the extent of involvement depends on the extent of nerve injury. Changes include central sensitization, neuroinflammation, and altered cortical representation. These changes explain the expansive list of symptomatology that many patients complain of after nerve injury. The central contribution to the clinical picture is not always clear, and whether these central changes are dependent on peripheral altered input or independently drive the clinical picture is not known. The extent of improvement in the patient’s symptomatology after surgical correction or local injection may help point to one approach versus another.

A double-crush injury phenomenon explains the point at which compression may not be bad but nerve dysfunction still occurs. The idea states that preexisting damage makes the nerve more vulnerable to injury from a subsequent insult. Similarly, pathology at the foraminal level makes carpal tunnel syndrome more likely, or preexisting carpal tunnel syndrome makes a person more likely to develop cervical radiculopathy symptoms. The latter is called reverse double-crush syndrome. These two phenomena are explained via disturbed axoplasmic flow, which is important for the health of the axon.

Stretching is another mechanism that contributes to work-related nerve injuries. Stretching of the nerve impairs blood flow. Altered conductivity in the nerve fiber is seen when blood flow is still above the ischemic threshold [39]. The nerves are more vulnerable to stretching compared to compression. Indeed, a 6% increase in length can lead to permanent damage [40].

Diabetes predisposes workers to entrapment neuropathy due to poorly functioning axonal transport systems, increased axonal swelling from a higher sorbitol concentration, and glycosylated collagen fibers that result in less compliant nerves [5].

## 5. Evaluation

The purpose of evaluation is to make an accurate diagnosis. This includes locating the site of damage, the extent of damage, and the mechanism of damage. When assessing work-related nerve damage, working conditions that predispose employees to injury are part of the evaluation. There are medical and legal connotations, with more legal and administrative implications than medical. The rules for evaluating work-related injuries are not unchangeable. An important aspect of the evaluation is immediate triage to determine whether surgical intervention is or is not needed, as delays worsen the prognosis. History is crucial. It is important to elicit the signs and symptoms of nerve dysfunction. Common presenting symptoms include pain, weakness, loss of function, numbness, tingling, and paresthesia. Insidious onset or vague complaints may delay the workup. History and physical examination are key components in localizing the site and extent of the pathology. Large myelinated fibers are more sensitive to pressure than small unmyelinated fibers [41]. This explains why pressure-based tests are used to evaluate entrapment neuropathies.

The Valleix phenomenon may be present, which is tenderness along the distribution of a peripheral nerve, both proximal and distal to the pressure-based entrapment [42]. Electro-diagnostic tests will confirm nerve pathology, the site, and the extent of dysfunction. These tests are helpful in proper clinical contexts. Early in an injury, electro-diagnostic tests may not reveal complete information, as the conduction component may still be normal if Wallerian degeneration has not set in. However, these tests are helpful in predicting prognosis and tracking recovery.

Electromyography is useful in evaluating loss of muscle innervation. The absence of any muscle activity—on recruitment and voluntary contraction—confirms complete nerve damage. No immediate spontaneous abnormal electrical activity (fibrillation waves) exists in the muscles as it takes several weeks to develop. The return of electrical activity with increased duration, complexity, and low amplitude with voluntary movement signifies re-innervation, which is a useful tracking tool. These electro-diagnostic tests are more effective in diagnosing chronic peripheral entrapment neuropathy as they can localize the site of entrapment. The predominant demyelination nature of chronic compression is easily revealed through conduction studies.

Imaging is beneficial in evaluating anatomies and searching for sources of compression. MRI and ultrasound are the most commonly used imaging modalities as they help in planning for surgical intervention. Imaging evaluates the causes of compression—e.g., soft tissue vs. bony etiology—and other pathologies that may be present. It can also evaluate effects of nerve damage by computing muscle mass or atrophic changes. MRI can show evidence of compression on the nerves as well as nerve flattening, swelling proximal to compression, and signal hyperintensity if edema is present. In addition, while ultrasound is more useful for injection therapy, its role in evaluating the etiology of compression continues to evolve [43].

An ultra-sonographic evaluation is preferred over MRI for distal entrapment neuropathies, largely due to its ease of use, access, cost-effectiveness, and ability to perform quick contralateral comparisons. Ultrasound technology has vastly improved the assessment of and intervention for musculoskeletal pain. It provides real-time assessment of muscles, tendons, and the condition of bones and nerves, all of which enables clinicians to make prompt and correct diagnoses. The use of ultrasound in evaluating nerve entrapment disorders is underappreciated. A high-resolution ultrasound can visualize individual nerve fascicles, sizes, and configurations and thus help diagnose various nerve pathologies [44]. It is a dynamic test that evaluates the diameter, shape, and ease of gliding a nerve between tissue planes. These are important characteristics when evaluating trapped peripheral nerves. The nerve diameter is key in diagnosing carpal tunnel syndrome or cubital tunnel syndrome [39].

## 6. Management

Treatment depends on etiology, duration, the extent of damage, and the severity of symptoms. Treatment options are both surgical and non-surgical. Conservative treatments avoid positions that further compromise nerve space and are aided by the use of splints. In addition, while physical therapy focuses on range of motion and manual therapy, its effectiveness remains limited. Nonsteroidal anti-inflammatory drugs (NSAIDs) are useful in the early phases of disease. Targeted steroid injections using ultrasound are effective in relieving pain in the short term; however, the level of evidence is low grade. In general, conservative interventions are short term, do not change underlying pathologies, and, at best, are symptomatic management of the disease. Most of all, they are based on insufficient evidence [45,46].

The outcomes of conservative interventions are difficult to ascertain as the natural course of compression neuropathy is variable. A lack of response to corticosteroid injections points to the non-inflammatory nature of the problem—as mentioned in the Pathophysiology section, chronic entrapment neuropathy is more like a degenerative-ischemic process. Surgical decompression is the definite treatment if symptoms persist (despite conservative management) or if motor weakness is evident. If addressed early, recovery is absolute. Delayed surgical intervention may not guarantee full recovery [47].

Development of entrapment neuropathy is multifactorial. Patient-dependent factors, such as genetics or biometrics, may not be modifiable, but improvement in any systemic disorder contributing to the disorder is equally if not more important in managing this condition. A number of work-related factors are associated with developing work-related entrapment neuropathies. For example, carpal tunnel syndrome is often associated with highly repetitive manual tasks that involve awkward hand or wrist postures, extreme or frequent flexion and extension of the hands, and forceful exertion or vibration of the hand/arm during work [48]. The American Conference of Government Industrial Hygienists (ACGIH) is a private, non-profit scientific association that publishes guidelines regarding the threshold limit value (TLV) for safe levels of exposure to biological, chemical, and physical agents in the workforce. The TLVs for these agents are published yearly [49].

The ACGIH employs a hand-activity level (HAL) assessment tool designed to evaluate the risk of work-related hand and musculoskeletal disorders (BSDs) of workers who engage in repetitive hand motion for 4 or more hours per day. Any activity above the TLV puts the worker at risk of developing musculoskeletal disorders; engineering or administrative measures should be taken to reduce the level of exposure. Kozal et al. found a positive correlation between the ACGIH HAL value and the risk of developing CTS [50]. Regular use of a splint and hand exercises are recommended, although the level of evidence remains low. Trillos-Chacón et al. systematically reviewed the literature on various strategies for preventing CTS in the workplace [51]. Among the interventions reviewed are a modification or change of accessories (keyboard, mouse, wrist rest, and workstations), education in ergonomics, exercise, and physical therapy. Unfortunately, the evidence is poor as most studies show a high risk of bias, primarily due to incomplete data, selective reporting of results, and insufficient blinding of researchers to make any strong recommendation.

## 7. Conclusions

Work-related peripheral neuropathies are common. The onset is generally insidious and multifactorial in nature. Clear causation is often lacking. In addition, while a better understanding of biomechanics for each case would be helpful, it is often lacking. Current management is focused on finding the extent and nature of compression and the magnitude of dysfunction. Compressive dysfunction is treated with surgical decompression, while non-compressive etiology is managed conservatively. Chronic pain is often a common outcome following chronic nerve compression. The outcome is not always optimal. This is due to an insufficient understanding of chronic compressive pathophysiology. Chronic compressive nerve injury is no longer considered a milder or protracted version of Wallerian degeneration. Macrophage infiltration and axonal damage are distinctly absent. The Schwann cell response is intense in the absence of any axonal damage. In the acute injury pattern, glial cells react to an acute axonal injury, while in the chronic compression pattern, Schwann cells respond before there is any injury to the axons. Schwann cells affect the function of neurons as opposed to an acute injury situation in which the axonal damage determines the Schwann cell response. It is likely that the Schwann cell response is triggered by pressure and/or chronic ischemia. The pressure-sensing mechanism of Schwann cells has not been elucidated. The focus is on the integrin signaling system as the system has been known to function as a bridge between extracellular mechanical stimuli (stretch, hydrostatic pressure, shear stress, and osmotic pressure) and intracellular signaling. A hydrostatic compression chamber model has been developed that will study the biological response of glial cells to a variety of physical pressures or stresses. An improved understanding of the myelination and remyelination mechanism, as seen in chronic compression injuries, will allow for the development of novel and more effective treatments. Schwann cells are regulated by a number of dedicated mechanisms, many known and many unknown, which, if well understood, can potentially be manipulated using pharmacological tools to amplify their support and repair role. This will go a long way in treating patients with acute as well chronic neuropathic conditions.

## Figures and Tables

**Table 1:** Incidence of the most common types of peripheral nerve entrapment in UK primary care [3].

	Men	Women
Carpal tunnel syndrome	87.8	192.8
Morton’s metatarsalgia	50.2	87.5
Ulnar neuropathy	25.2	18.9
Meralgia paresthetica	10.7	13.2
Radial neuropathy	2.97	1.42

Per 100,000 people.
